# Biologic Therapy in HIV: To Screen or Not to Screen

**DOI:** 10.7759/cureus.15941

**Published:** 2021-06-26

**Authors:** Willam Davis, Ilan Vavilin, Nidhi Malhotra

**Affiliations:** 1 Internal Medicine, Georgetown University, Washington, DC, USA; 2 Gastroenterology, Washington Hospital Center, Washington, DC, USA

**Keywords:** biologic therapy, ustekinumab, hiv, aids, screening, crohn’s disease (cd), inflammatory bowel disease

## Abstract

Biologic therapy has revolutionized the management of chronic inflammatory diseases, including inflammatory bowel disease (IBD). Before the initiation of therapy, it is critical to rule out prior infection of chronic hepatitis B virus (HBV) and tuberculosis (TB). However, screening for human immunodeficiency virus (HIV) is not as routinely completed. We present a case of a 56-year-old male on ustekinumab for the management of Crohn’s disease, found to have undiagnosed human immunodeficiency virus (HIV) with progression to acquired immunodeficiency syndrome (AIDS).

## Introduction

The use of biologic therapy is becoming the standard of care treatment in IBD, especially in Crohn’s disease [1,2]. Routine testing for chronic HBV and TB is recommended due to the risk of disease reactivation during the initiation and maintenance therapy [3-5]. However, the recommendation for HIV screening has evolved in the past decade [3-5]. This modification in screening guidelines holds particular importance in individuals at high-risk of contracting HIV, as further immunosuppression may place this population at higher risk for opportunistic infections. We want to emphasize the importance of HIV screening with the case of a 56-year-old man on ustekinumab for the management of Crohn’s disease, found to have undiagnosed HIV with progression to AIDS. 

## Case presentation

**Figure 1:**
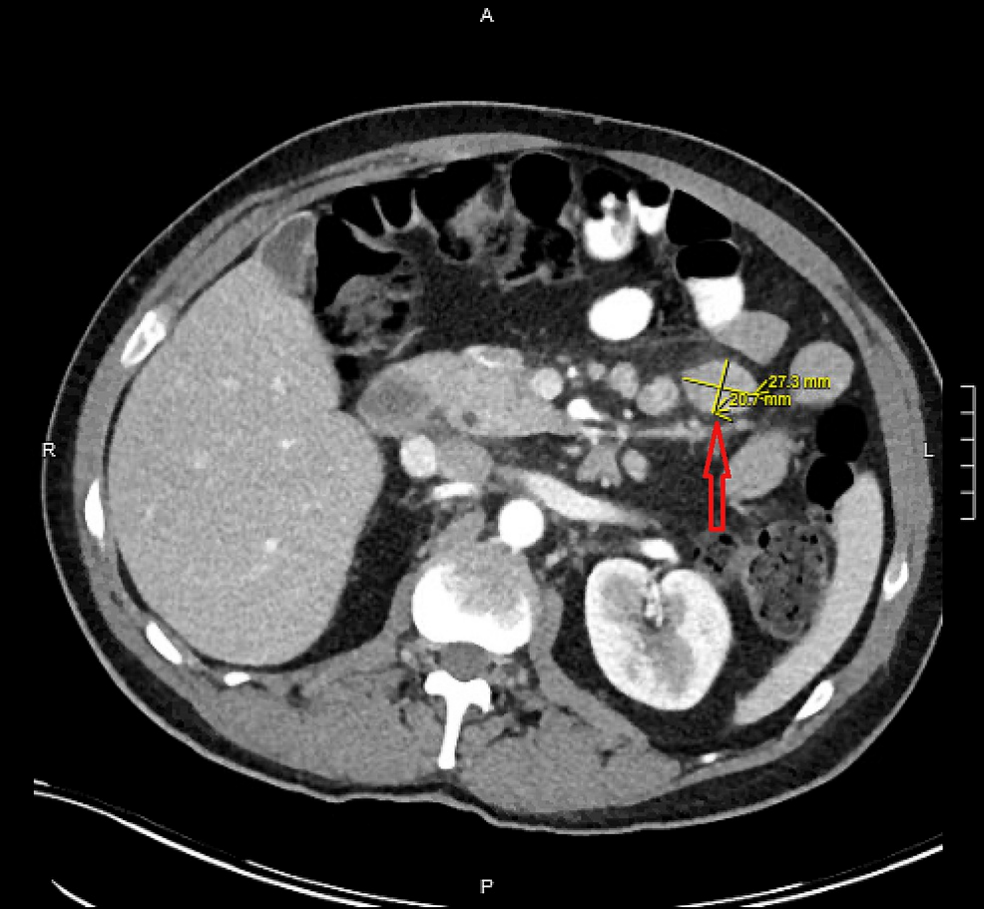
CT abdomen pelvis demonstrated enlarged mesenteric lymph node (red arrow), largest measuring 2.7x2.1 cm (yellow markings).

**Figure 2:**
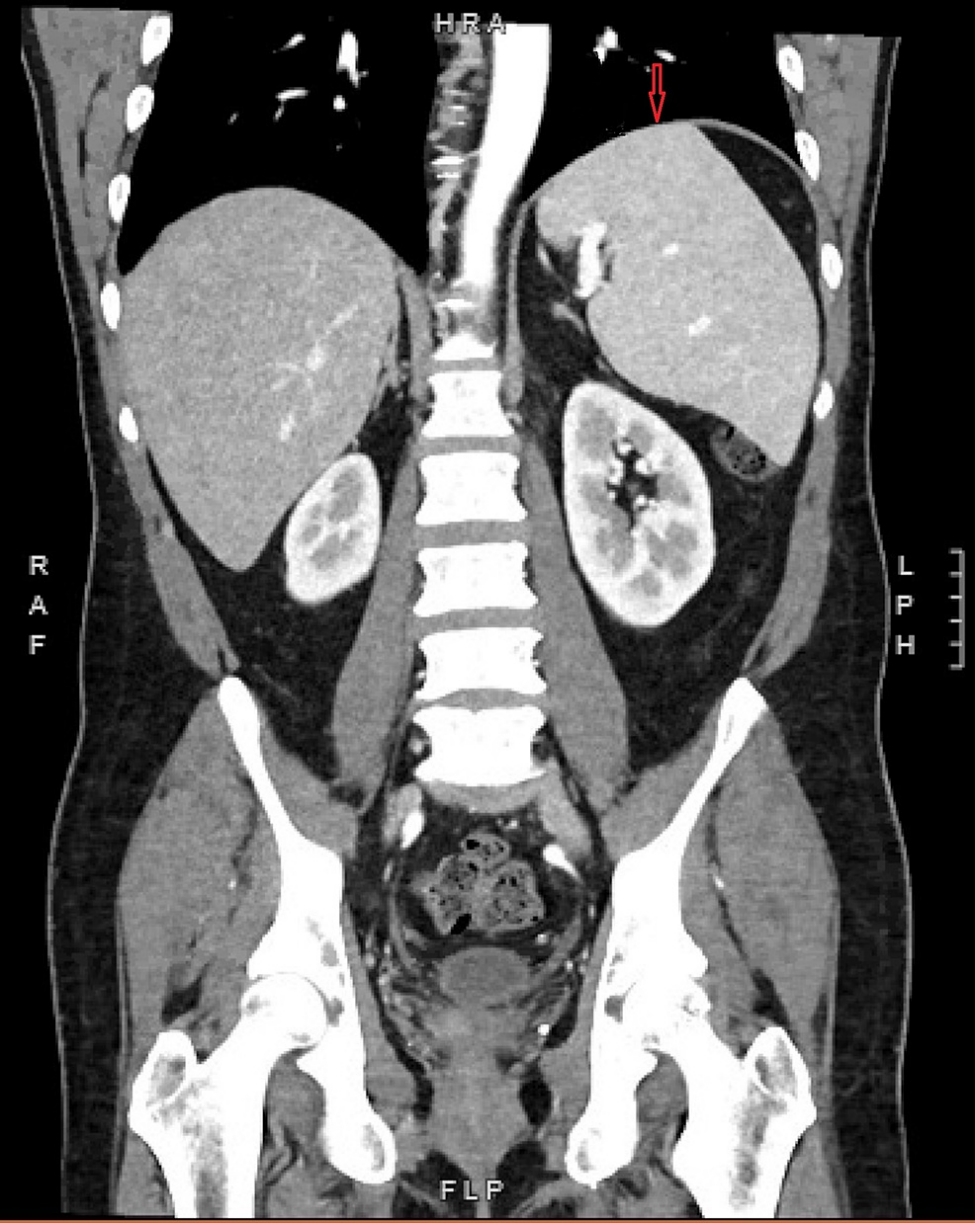
Coronal plane CT of the abdomen and pelvis demonstrating splenomegaly (red arrow) measuring 15.2 cm craniocaudally

The 56-year-old male had a medical history of same-sex relationships with men, recurrent salmonella urinary tract infections (UTIs), Crohn's disease, and on ustekinumab therapy for the past two years, and was hospitalized for abdominal pain, early satiety, and weight loss. On admission, the physical exam showed a man who appeared to be chronically ill with bitemporal wasting, oral mucosa without evidence of oral thrush or ulcerations, and pulmonary crackles heard on the lung auscultation. The lab results were remarkable, including a leukopenia of 1.6x103/μL, with an absolute neutrophil count of 0.8x103/μL and an absolute lymphocyte count of 0.3x103/μL. The computerized tomography (CT) scan of the abdomen and pelvis with IV contrast showed enlarging mediastinal and mesenteric lymph nodes with splenomegaly (Figure 1-2).

**Figure 3:**
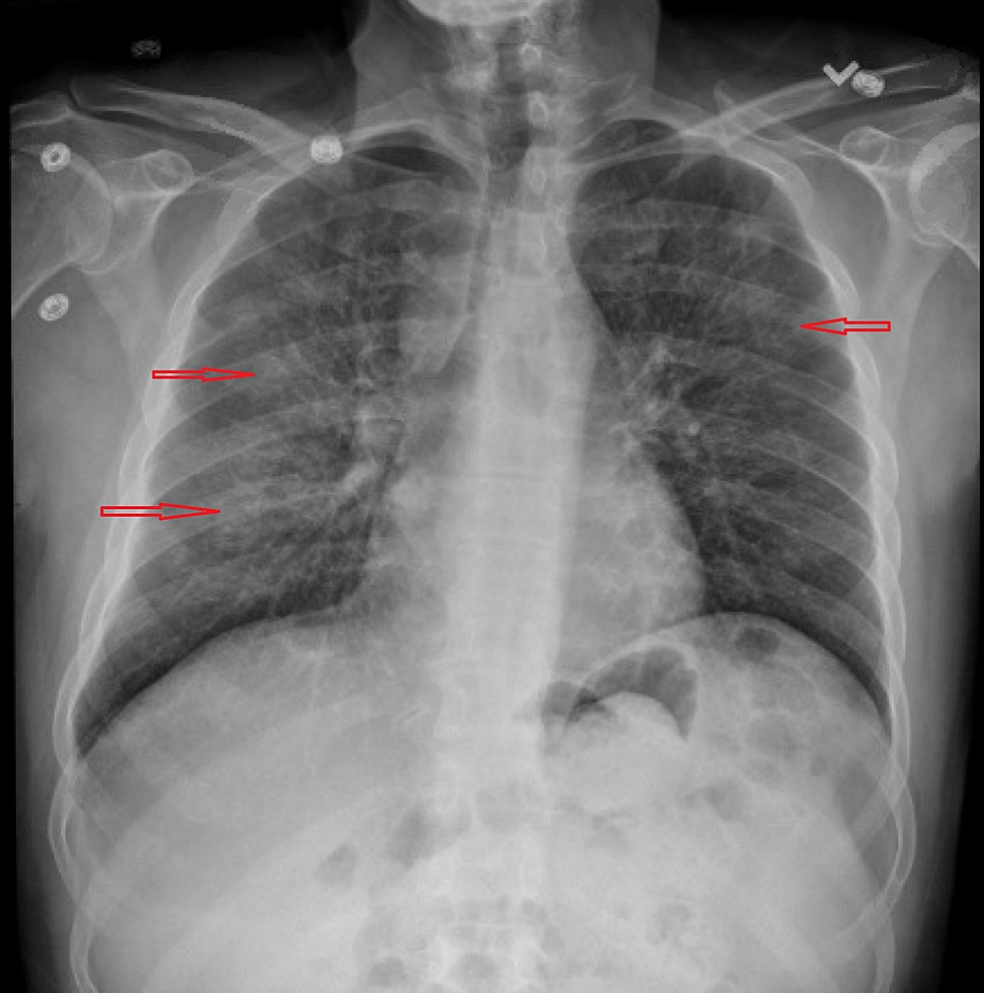
Chest X-ray demonstrating mild diffuse hazy opacities (red arrows) throughout both lung fields

**Figure 4:**
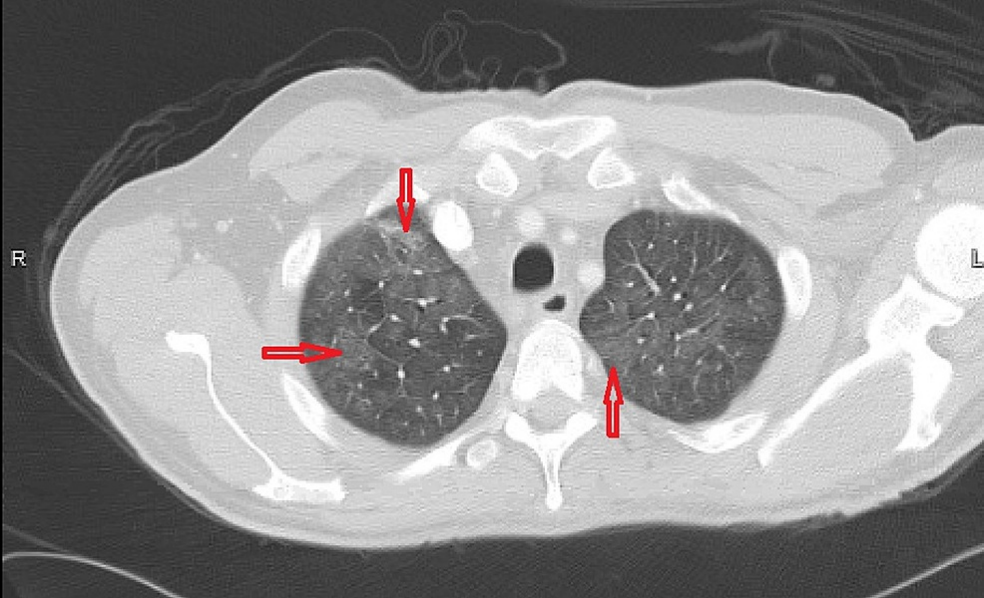
CT chest scan with IV contrast demonstrating moderate diffuse ground glass opacities (red arrows)

In the setting of neutropenia, the findings were concerning for lymphoma. An extensive workup was undertaken for the lymphadenopathy including histoplasmosis, tuberculosis, SARS-CoV2 which tested negative. Serum PCR was positive for Epstein-Barr virus of 1191 IU/ml and cytomegalovirus of 908 copies/ml. The patient additionally was found to be HIV-1 positive, with an absolute CD4 count of 11/μL and a viral load of 2,650,000copies/mL. Trans-bronchial biopsies of a mediastinal lymph node revealed granulomatous changes without the evidence of lymphoma. The patient’s ustekinumab was discontinued and he was prescribed prednisone, primaquine, and clindamycin for suspected pneumocystis pneumonia based on the chest X-ray and CT chest findings (Figures 3-4). He was continued on cefdinir for a chronic salmonella UTI. The patient was subsequently discharged to be followed up on his infectious disease and to initiate him on highly active antiretroviral therapy (HAART) as an outpatient. 

The subject of our case study was diagnosed with Crohn’s disease in 2018. At that time, he was not immune to HBV and completed a course of hepatitis B vaccinations. The QuantiFERON-TB Gold test proved negative. At the time of diagnosis, old fistulous sites were noted endoscopically. Additionally, segmental areas of severely congested, erythematous, plaque-covered, and vascular-pattern-decreased mucosa with aphthous and deep ulcers were found in a non-contiguous pattern throughout the rectum and colon. He was subsequently started on ustekinumab in 2019.

The patient has had four episodes of recurrent non-typhi salmonella UTIs, documented with a positive urine culture. An extensive workup for recto-vesical fistula was negative, including a magnetic resonance enterography, CT with rectal contrast, as well as a cystoscopy. While he was reportedly tested for HIV 10 years prior, no results were available in the electronic medical records.

## Discussion

Biologic therapy is pivotal in the management of moderate to severe IBD, especially in Crohn’s disease [1,2]. Due to the immunosuppressant effects of these medications, patients are at risk of the reactivation of chronic infections, in particular HBV and TB [6]. The European Crohn’s and Colitis Organisation (ECCO) provides an updated, evidence-based consensus on screening recommendations for opportunistic infections in IBD patients receiving biologic therapy [3-5]. Screening for HBV and TB prior to initiating and routinely during biologic therapy has been well established in the literature [3-5]. With regards to HIV, ECCO has adjusted its screening guidelines over the past decade. The recommendations changed from “should be considered” to “recommended” in 2014 [3,4]. The ECCO 2014 guidelines also recommended re-testing for HIV among high-risk populations for which the Centers for Disease Control and Prevention (CDC) recommends at least an annual screening for those at increased risk [4,7]. The World Health Organization defines high-risk factors as same-sex relationships in men, the use of contaminated needles, and concomitant sexually transmitted diseases [8]. In HIV-positive patients, the updated ECCO 2021 guidelines recommend achieving a stable CD4 count and an undetectable viral load on HAART prior to beginning immunosuppression [5]. These patients did not appear to be at an increased risk of opportunistic infections from immunosuppression [5].

We are not aware, following our thorough literature review, of controlled trials of biologic therapy in the HIV population, as these patients are typically excluded. However, its safe use among HIV patients is well documented through multiple case reports and case series of HIV patients with psoriasis receiving biologic therapy [9].

Psoriasis in patients with HIV is often severe and extremely difficult to manage [9]. Initial therapy consists of HAART. However, many patients often require chronic management with biologic therapy [9]. A review completed by Bartos et al of 27 HIV-positive patients with psoriasis found that 24 out of the 27 patients were on HAART, of which four out of 24 were treated with ustekinumab [10]. The review concluded that patients treated with ustekinumab, adalimumab, etanercept or infliximab tolerated biologic therapy with stable to improved viral loads and CD4 counts. One exception was a patient treated with ustekinumab that had a drop in CD4 from 523cells/mm^3 to 454cells/mm^3 [10]. Based on this review and ECCO recommendations, biologic therapy in HIV-positive patients may be safe in the setting of the HAART-controlled HIV disease. However, future clinical trials should be directed to further support this finding.

For this case study, the 56-year-old patient had been screened ~10 years ago for HIV. Taking into account the CDC and ECCO’s recommendations for the re-testing of high-risk individuals, this patient should have received an updated HIV screening prior to starting him on ustekinumab, and at least yearly thereafter. Additionally, his history of recurrent non-typhi salmonella UTIs should have raised suspicion for underlying HIV [11]. Due to severe T-cell function and macrophage phagocytosis impairment, HIV-infected patients are at a particularly high risk of developing severe and recurrent salmonella infections [11]. Moreover, in an HIV-positive individual, recurrent salmonella bacteremia is considered an AIDS-defining illness [11].

## Conclusions

With no prior diagnosis of HIV and subsequent follow-up testing, it cannot be determined if disease progression to AIDS was accelerated by the further immune compromising effects of biologic therapy or the natural course of untreated HIV. Nonetheless, based on the literature guidelines of ECCO, all patients should receive screening for HIV before starting biologic therapy. Identifying the HIV-positive population will allow for the initiation of HAART treatment and promote viral suppression prior to biologic therapy. In doing so, it will decrease the risk of further immune suppression and opportunistic infections.
